# Psychometric Properties of Suboptimal Health Status Instruments: A Systematic Review

**DOI:** 10.3390/jpm13020299

**Published:** 2023-02-08

**Authors:** Mohamed Ali Alzain, Collins Otieno Asweto, Sehar-un-Nisa Hassan, Mohammed Elshiekh Saeed, Ahmed Kassar, Bandar Alsaif

**Affiliations:** 1 Department of Public Health, College of Public Health and Health Informatics, University of Ha’il, Ha’il 55476, Saudi Arabia; 2 Department of Community Medicine, Faculty of Medicine and Health Sciences, University of Dongola, Dongola 41111, Sudan; 3 Department of Community Health, School of Nursing, University of Embu, Embu 6-60100, Kenya; 4 Faculty of Medicine, National University-Sudan, Khartoum 11115, Sudan; 5 Department of Physiology, Faculty of Medicine, University of Dongola, Dongola 41111, Sudan

**Keywords:** sub-optimal health status, measurement, tools, instruments, psychometrics, reliability, validity, a systematic review

## Abstract

Background: Suboptimal health status (SHS) measurement has now been recognized as an essential construct in predictive, preventive, and personalized medicine. Currently, there are limited tools, and an ongoing debate about appropriate tools. Therefore, it is crucial to evaluate and generate conclusive evidence about the psychometric properties of available SHS tools. Objective: This research aimed to identify and critically assess the psychometric properties of available SHS instruments and provide recommendations for their future use. Methods: Articles were retrieved by following the guidelines of the PRISMA checklist, and the robustness of methods and evidence about the measurement properties was assessed using the adapted COSMIN checklist. The review was registered in PROSPERO. Results: The systematic review identified 14 publications describing four subjective SHS measures with established psychometric properties; these included the Suboptimal Health Status Questionnaire-25 (SHSQ-25), Sub-health Measurement Scale Version 1.0 (SHMS V1.0), Multidimensional Sub-health Questionnaire of Adolescents (MSQA), and the Sub-Health Self-Rating Scale (SSS). Most studies were conducted in China and reported three reliability indices: (1) the internal consistency measured by Cronbach’s α value ranged between 0.70 and 0.96; (2) the test–retest reliability; and (3) the split-half reliability coefficient values ranged between 0.64 and 0.98, and between 0.83 and 0.96, respectively. For the values of validity coefficients in the case of SHSQ-25 > 0.71, the SHMS-1.0 ranged from 0.64 to 0.87, and the SSS ranged from 0.74 to 0.96. Using these existing and well-characterized tools rather than constructing original tools is beneficial, given that the existing choice demonstrated sound psychometric properties and established norms. Conclusions: The SHSQ-25 stood out as being more suitable for the general population and routine health surveys, because it is short and easy to complete. Therefore, there is a need to adapt this tool by translating it into other languages, including Arabic, and establishing norms based on populations from other regions of the world.

## 1. Introduction

Health has been traditionally conceptualized as a biological function at typical levels of efficiency [1]. However, health, as defined by the WHO, is not just the absence of sickness, but rather the presence of complete well-being [2]. As chronic diseases rise, several researchers suggested redefining health. Health is the ability to adjust to social, physical, and emotional obstacles [3]. In some instances, the medical literature also mentions a third status, which is described as non-disease and non-healthy [3]. Several states of physical discomfort and signs that could not be clearly described as diseases were listed in the ICD-10. These vague health conditions were ascribed as ‘sub-optimal health status’ (SHS) by scholars; later, in 2009, a team of Chinese researchers developed a tool to measure this health status [4]. The construct of SHS has been described as a sub-clinical state where a person is neither experiencing sickness nor is healthy, and there are apparent signs of discomfort [5], which may lead to adverse health outcomes [6]. Since the definition of health has several implications for health policy, practices, and healthcare services, it is essential to define and appropriately measure various states of health and well-being. Recently, scholars and clinicians have pointed out that the current definition of health by the WHO needs to be revisited, in order to deal with emerging challenges in the health system, lifestyle factors, and environmental issues that impact the health and well-being of individuals [7].

SHS has been conceptualized by Feng [8] under the health model, which encompasses biological, psychological, and social domains of health. Low quality of health status without any apparent disease condition experienced by individuals in health domains is considered a sub-health condition. In the physical domain, it presents itself as poor functioning of the body and organs, along with diminished energy levels; deprivation of emotional and cognitive resources for functioning relates to the psychological domain; and in the social domain, it is depicted by non-availability or poor utilization of social resources that may hamper some aspects of social functioning.

Many developed countries, including Saudi Arabia, are undergoing vivid shifts due to fast-paced economic growth, which impacts lifestyles and social systems. The literature from other high-income countries has pointed out that unhealthy lifestyles significantly impact health, and mainly increase the vulnerability for sub-optimal health status [9]. Among the reasons, currently, both a high number of men and women in the workforce are exposed to work pressures and risk for work–family imbalance, and the COVID-19 epidemic has had a negative effect on people’s standard of living in several domains and may have increased the risk of developing SHS [10,11].

SHS is a heightened concern for medical professionals and public health experts because it is a significant risk marker for chronic illnesses. SHS is differentiated from a sub-clinical disease state because it is a low-quality health state that cannot be classified as a disease state [3]. The signs and symptoms experienced by individuals at the onset of mental and psychological disorders have proximity to symptoms of SHS and must be differentiated thoroughly. SHS is usually demonstrated by deterioration in physiological, emotional, and social functioning, leading to a decline in vitality, adaptation, and resilience. According to the diagnostic guidelines provided by the Association of Chinese Medicine [12], symptoms in three areas, namely, systematic, psychological, and social, are evaluated to assess SHS. Among core physical and psychological symptoms are body aches and pain, tiredness, disturbed sleep, low mood, irritability, restlessness, reduced focus, and memory problems. Individuals also experience a decline in interest and engagement in social activities. The decision to diagnose SHS in any three dimensions is made, if an individual experiences symptoms over the previous three months without a baseline disease condition. However, this approach was less accepted due to its subjectivity and was not employed in clinical diagnosis [13]. 

Different quantitative, qualitative, and mixed approaches have been used to measure SHS [14,15]. Among the quantitative measures, self-rating scales and checklists have been commonly used, among which are the Suboptimal Health Status Questionnaire- 25 (SHS-25) [4,16] and the sub-health Measurement Scale V1.0 [17]. The comprehensive assessment of SHS also includes measuring stress response using biochemical methods; stress response is considered a causal mechanism that increases the risk of experiencing SHS [18]. Furthermore, a study demonstrated that people with SHS were more likely to report symptoms of fatigue and pain [19]. There are two categories of measures used in determining the SHS. Among the objective indicators are biochemical and anthological-physiological indicators, such as C-reactive protein (CRP), low-density lipoprotein (LDL), high/low blood pressure (BP), and high/low body mass index (BMI). In current medical practice, subjective measures are employed for the clinical diagnosis of SHS after a comprehensive physical examination that excludes specific illnesses. In public health research, self-report measures are widely used to assess SHS.

Interestingly, there are also controversies about whether the measurement of SHS aligns with the SHS theoretical framework, which assumes that environmental factors and psychological states determine SHS, and betterment in these factors should alleviate SHS [16]. Therefore, SHS is seen as a reversible health condition, compared to disease conditions that have progressed toward worsening symptoms.

SHS has now been recognized as an essential construct in personalized medicine to decrease the risk of developing disease and enhance general health. Moreover, the idea of SHS reflects the belief that chronic diseases can be effectively predicted and prevented before a clinical manifestation of severe pathologies from the view of predictive, preventive, and personalized medicine [3,5]. It is crucial to have reliable tools to assess SHS, which can be used in clinical practice and community health research. We have noticed that there are limited tools, an ongoing debate about the definition and measurement of SHS, and inconclusive evidence about the psychometric properties of tools to measure SHS [20,21]. Despite the diagnostic standards and assessment of SHS having been shifted to objective indicators, there are unresolved issues related to the appropriateness of measures used in assessment due to the wide range of symptoms, the intensity of symptoms experienced, and their link with many diseases’ conditions [20,21,22]. 

Appropriate measurement of SHS is vital to designing effective community health interventions. Therefore, the purpose of this article is to answer the following research questions:

- What instruments are available to measure SHS for different segments of populations?
- What are the key strengths and weaknesses of measures used to assess SHS in target populations?
- To what extent have the psychometric properties of these SHS instruments been evaluated for use in target populations?
- What are the current gaps in generating conclusive evidence about the psychometric properties of these tools in different populations?

Given that self-report measures are widely used to assess SHS, practitioners and researchers need validated and reliable self-report instruments that can easily be administered to assess SHS. Therefore, it is essential to conduct a systematic review of the psychometric properties and utility of subjective SHS measures to guide in selecting appropriate SHS instruments for health research and clinical assessment. However, the currently available systematic reviews focused on the construct and conceptual framework of SHS [3,5] to date; there is no systematic review that has summarized the psychometric properties of tools used in the assessment of SHS (Table 1). Thus, we aimed to identify and critically assess the psychometric properties of available SHS instruments and provide recommendations for their future use. The scope of the current study matches the most recent recommendation made regarding the vigour of SHS instruments in prognostic, preventive, and personalized medicine [23]. 

## 2. Materials and Methods

A systematic review of global studies was conducted to assess the measurement properties and robustness of scales used to assess SHS. In this study, we followed the guidelines of the Centre for Reviews and Dissemination [23] and preferred reporting items for systematic review and meta-analyses protocols (PRISMA-P) [25]. We devised our search strategy and filters by the Consensus-based Standard for the Selection of Health Measurement Instruments (COSMIN), because it is an effective way to conduct a thorough and systematic review of health measures [26]. The review was registered in the PROSPERO international prospective register of systematic reviews (registration number CRD42021290565). 

### 2.1. Search Strategy and Filters

The term suboptimal health status was first coined in 2002 by a Chinese researcher, as cited in [15], and the research on the assessment of SHS using objective measures also began in the same year, as reported in a systematic review of SHS that was conducted in 2015 [3]. Therefore, we limited our search to original articles that were published between Jan 2002 to Jan 2022, and available on any of the four electronic databases, namely the Web of Science (WOS), Scopus, PubMed, and Embase. 

By the Consensus-based Standard for the Selection of Health Measurement Instruments (COSMIN) [27], we searched articles in four areas: ‘construct search’, ‘population search’, ‘instrument search’, and ‘measurement properties. A search strategy was performed using the terms MeSH “suboptimal health status”, “health status”, “instruments”, “survey”, “scale”, “adult”, “men”, “women”, “adults”, “elderly”, “validation studies”, “surveys”, “outcome measures”, “psychometrics”, “internal consistency”, and other relevant search filters. The exclusion filter was applied to remove irrelevant records from the search, such as case reports and animal studies. The details of all the filters and terms used are available in Flowsheet Diagram 1 and Supplementary Files S1 and S2.

**Figure 1:**
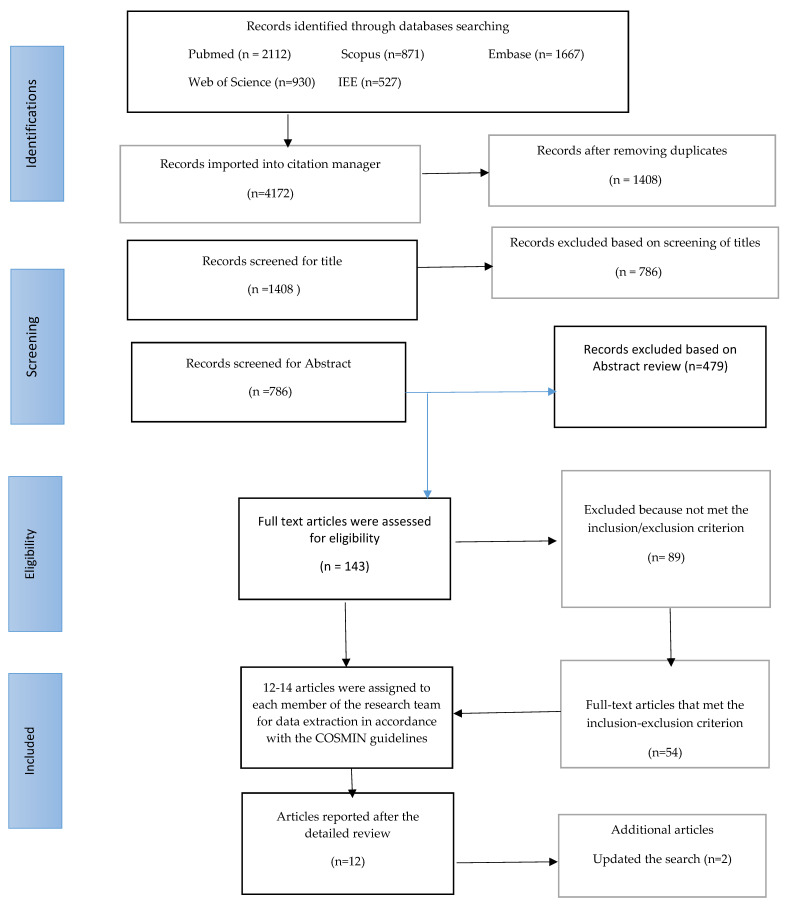
PRISMA (Preferred Reporting Items for Systematic Reviews and Meta-Analyses) flow chart.

This search strategy and filter were applied in an original article search on each database. The article search was completed by three (MAA, COA, and SuNH) out of six investigators on this project, from January 2022 to February 2022. It was limited to articles published between Jan 2003 and Jan 2022 in the English language only. The inclusion and exclusion criteria for determining the eligibility of articles were as follows: (1) original studies on the measurement of SHS; (2) conducted with adult and adolescent populations; (3) studies undertaken in the last 20 years (Jan 2002 to Jan 2022); (4) studies complying with ethical standards such as the Declaration of Helsinki codes. Studies exclusively focusing on evaluating clinical interventions, systematic reviews, and meta-analyses were not included. This search yielded over 4500 research articles from each database, and the Microsoft Excel and CVS files from the databases were downloaded for the initial inspection of datasets. This MS Excel file contained basic identifying information about the articles, such as authors’ names, titles, publication dates, journal names, Doi numbers; then, an identification number was allotted by the database to each article. This basic information was used to remove the duplicated records, and a list of final articles was finalized for initial review by the researchers against the eligibility criteria. The article search, retrieval, and selection steps are presented in the flowsheet diagram (Figure 1).

### 2.2. Evaluation of Articles

In the first stage, two researchers (MAA and COA) carefully read the article titles and abstracts to filter the articles that match the eligibility criteria. Where doubts arose regarding inclusion, the method section of the article was read to access details. This search revealed that 54 articles met the inclusion/exclusion criterion. In the second stage, about 12 to 14 articles were assigned to four investigators on the project (ASA, SuNH, MAA, and COA), who read complete articles and filled in a data extraction form prepared by the research team containing questions based on The Consensus-based Standards for the Selection of Health Measurement Instruments (COSMIN) checklist to assess the procedural diligence and main findings related to measurement properties of the instruments [26,27]. In the final stage, 14 studies were included for detailed review in accordance with the checklist that assessed the measurement properties of tools along with other information on the studies, which includes (a) Name of the instrument/measure of SHS: (b) author(s) name/publication year/study region/country; (c) study sub-population (young/adults /men/women/elderly), sample size and characteristics (d) population with non-communicable diseases excluded vs included (e) study methods: quantitative vs qualitative, data collection methods and study procedure; (f) psychometric properties: forms of validity and reliability determined in the study and any other main findings. 

### 2.3. Methodological Quality Assessment

The study’s methodological quality was assessed using the adapted version of the COSMIN Risk of Bias checklist manual for systematic reviews of PROMs [26]. We assessed the studies on some indicators of reliability and validity that include (a) internal consistency; (b) test-retest reliability; (c) structural validity; (d) convergent and discriminant validity; (e) indices of the factor structure, as shown in the flowsheet diagram (Figure 1).

## 3. Results

This systematic search retrieved four subjective measures for assessing SHS in adults, adolescents, and university students. These included the following: (1) Suboptimal Health Status Questionnaire- 25 (SHSQ-25), (2) Sub-health Measurement Scale Version 1.0 (SHMS V1.0), (3) Multidimensional Sub-health Questionnaire of Adolescents (MSQA), and (4) Sub-Health Self-Rating Scale (SSS) [5,23].

The psychometric properties of these tools were assessed through some reliability and validity indicators, as shown in Table 2 and Table 3.

### 3.1. Suboptimal Health Status Questionnaire-25 (SHSQ-25)

#### 3.1.1. Description of the SHSQ-25

The SHSQ-25 is the most commonly used SHS screening tool. China’s Capital University of Medical’s Wei Wang group invented it in 2007 [3,4], and readily articulated and operationalized it in 2009 [28]. The questionnaire is the outcome of a focus group discussion with apparently healthy individuals, an extensive literature search, and expert opinions [4]. The SHSQ-25 was developed to screen and take into account multidimensional health constructs that could indicate people were feeling poor health and acquired chronic stress. The authors formulated a questionnaire containing 25 items in five domains: (1) fatigue (9 items), (2) the cardiovascular system (3 items), (3) the digestive tract (3 items), (4) the immune system (3 items), and (5) mental status (7 items) [3,4,15,24,28]. It assessed how often individuals suffered from several specific discomforts in the previous three months [24].

The SHSQ-25 is rapid and easy to complete; therefore, it is appropriate for the general population and healthcare settings [4,13,15]. It has been applied and validated in various populations, including Chinese, African, and European [23]. Despite widespread applications of the SHSQ-25, most studies explore psychometric properties only in the Chinese population and, recently, in Ghanaian and Korean populations [28,29,30].

#### 3.1.2. Scoring System for the SHSQ-25

SHSQ-25 items are scored on a 5-point Likert scale, from never to always [5,19,31,32]. The total score is the aggregate of all 25 questions, scored from 0 to 4 [4,5,14,16]. SHS screening uses the upper limit of a unilateral 90% reference value (X + 1.28S), if the population’s SHS score follows a normal distribution [4]. The percentile and the unilateral P90 value’s upper limit will be used if it does not follow a normal distribution [4]. The SHSQ-25 considers all important factors that affect SHS; hence, the cut-off point is 35 points, the highest limit of the unilateral P90 value [4].

#### 3.1.3. Reliability and Validity Indicators of the SHSQ-25

We found four studies assessing the instrument’s internal consistency and test–retest reliability. Yan et al. (2009) examined 3000 Chinese individuals, and found item-sub-scale correlations ranging from 0.51 to 0.72, and a Cronbach’s α 0.93 for all sub-scales [4]. Furthermore, Wang and Yan (2012) [15] found a higher Cronbach’s α value (0.91) for internal consistency among 3045 Chinese individuals. Interestingly, Adua, et al. (2021) [28], while assessing the internal consistency of SHSQ-25 among 263 healthy Ghanaians, found Cronbach’s α for each category as follows: fatigue = 0.846, immune-cardiovascular = 0.820, and cognitive = 0.864 [28]. Only one study had test–retest reliability with coefficient values of 0.89 to 0.98 [4]. Adua, et al. (2021) assessed the validity, and the findings revealed a construct validity > 0.7 thresholds [28]. Meanwhile, convergent and discriminant validity values were as follows: fatigue (AVE = 0.366, MSV = 0.701), cognitive (AVE = 0.358, MSV = 0.671), immune-cardiovascular (AVE = 0.537, MSV = 0.185. Recently, a study validated the Korean Septimal Health Questionnaire (KSHSQ-25), and the findings revealed that the test–retest reliability’s range was 0.88–0.99, a Cronbach’s α of 0.953, and a Cronbach’s α for each domain ranged from 0.76 to 0.94 [30], as shown in Table 2.

### 3.2. Sub-Health Measurement Scale V1.0 (SHMS V1.0)

#### 3.2.1. Description of the SHS V1.0

The second instrument was the Sub-Health Measurement Scale V1.0. It is a self-reported multidimensional inventory designed to assess physiological, psychological, and social symptoms to determine SHS. This inventory was devised by researchers in China [31]. The inventory consists of a total of 39 items. The first four items are used to evaluate individual general health, and the remaining thirty-five items are divided into three dimensions [30]. The dimension of physiological symptoms encompasses four factors, which are physical condition, organ function, body movement function, and vigor; these factors are assessed through a set of fourteen questions [33]. The psychological dimension of symptoms contains three factors, which are positive emotions, psychological symptoms, and cognitive functions, and are assessed through a set of twelve questions [30]. The social dimension includes three factors, which assess social adjustment, resources, and support, and comprises a set of nine questions.

The SHMS V 1.0 has been widely used to determine the SHS of participants, especially among nurses, urban residents, college students, and midwives [9,32,34,35,36,37]. The scale was proven to have good psychometric properties in these studies [31,38,39,40].

#### 3.2.2. Scoring System for the SHMS V1.0

The SHMS V1.0 has a straightforward scoring system. On a five-point Likert scale, from 1 (never) to 5 (often), respondents are asked to rate how often they experienced various types of discomfort over the past six months [33]. A set of items comprises three dimensions, and the total sub-score sums up the score on each dimension. The transformed score is computed using the score conversion formula. The converted score lies between 0 and 100, and represents the health status [33]. A lower total score is interpreted as a worse health status. The cut-off scores are used to differentiate between individuals with positive health and SHS on all three dimensions [41]. These are 66.1, 52.1, and 55.6 for physiological, psychological, and social dimensions, respectively [36]. If the score for these three dimensions is found to be lower than the cut-off, the participant is categorized as having physiological, psychological, and/or social health SHS. In another study [39], the mean, percentile, and threshold norms were established. According to sex and age brackets (14–19, 20–29, 50–64, and 65), norms for the total, physical, mental, and social sub-health of Chinese urban residents were calculated. Computing the mean ± SD and mean ± 0.5SD of the transformed scores yields the threshold norms of SHMS V1.0’s five health states: illness, severe SHS, moderate SHS, mild SHS, and positive health [41].

#### 3.2.3. Reliability and Validity Indicators of the SHMS V 1.0

We found five studies assessing SHMS V 1.0 psychometric properties; the structural validity showed a high correlation between an item and dimensional scores (0.656 to 0.878). The correlation between each dimension and sub-scale scores was strong (0.586 to 0.868) [42]. We found that the reliability of the SHMS V 1.0 was 0.917. The first study’s Cronbach α coefficient was 0.92, and the split-half coefficient was 0.83 [31]. A recent study in Tianjin found that the test–retest and overall Cronbach’s coefficients were 0.67 and 0.92, respectively. In addition, the correlation between the SHMS v1.0 and SF-36 was 0.78 (*p* < 0.01) [40], as indicated in Table 2.

**Table 2:** Studies on reliability and validity of SHSQ-25 and SHMS V 1.0 instruments.

Instrument	Authors and Year	Sample Size	Reliability		Validity
			Internal Consistency	Test–Retest Reliability	
SHSQ-25	Yan et al. (2009) [4]	3000 adults	Cronbach’s α 0.93, item-sub-scale correlations 0.51–0.72	Range (0.89–0.98)	Content validity KMO = 0.93 Bartlett test < 0.001
	Wang and Yan (2012) [15]	3405 individuals	Cronbach’s α = 0.91	Not Reported	Not Reported
	Adua, et al. (2021) [28]	263 healthy adults	Cronbach range 0.7–0.9 Cronbach’s α for sub-scales: Fatigue = 0.846, Immune-cardiovascular = 0.820, Cognitive = 0.846	Not Reported	Construct validity > 0.7 thresholds Convergent and discriminant validity on sub-scales: Fatigue (AVE = 0.366, MSV = 0.70) Cognitive (AVE = 0.358, MSV = 0.67). Immune-cardiovascular (AVE = 0.537, MSV = 0.185).
	Guo, Z. et al. (2022) [30]	460 healthy adults	Cronbach’s α of 0.953 and Cronbach’s α for each domain ranged from 0.76 to 0.94	Range (0.88–0.99)	Root mean square error of approximation (RMSEA) = 0.069 < 0.08 Adjusted goodness of fit index (AGFI) = 0.907 > 0.90
SHMS V 1.0	Xu et al. (2011) [31]	2000 individuals	Cronbach α = 0.92, split-half reliability = 0.83	0.64	Correlation coefficient between SHMS V1.0 and SF-36 was 0.664 (*p* < 0.001).
	Wu et al. (2016) [38]	24,475 individuals	Cronbach’s α = 0.91, Cronbach’s α for three dimensions range 0.82–0.85	Not Reported	Not Reported
	Lolokote et al. (2017) [36]	829 college students	Cronbach’s α = 0.89, sub-scale alpha range 0.71–0.85	Not Reported	Not Reported
	Ma et al. (2020) [41]	5233 university students	Cronbach’s α = 0.68	Not Reported	Not Reported
	Miao et al. (2021) [40]	2640 individuals	Cronbach’s α = 0.92, Cronbach’s α on sub-scales: Physical sub-health = 0.85, Psychological sub-health = 0.87, Social adaptation sub-health = 0.89	0.67	Correlation between dimensional range between 0.65 and 0.87 Correlation between SHMS v1.0 and Short Form-36 (SF-36) = 0.67 Correlation between SHMS v1.0 and SF-36 was 0.78

AVE: average variance extract; MSV: maximum shared variance; SHSQ-25 = Suboptimal Health Status Questionnaire-21; SHMS V 1.0 = Sub-health Measurement Scale Version 1.0; KMO = Kaiser–Meyer–Olkin.

### 3.3. Multidimensional Sub-Health Questionnaire of Adolescents (MSQA)

#### 3.3.1. Description of the MSQA

Our systematic search found the MSQA, an adolescent assessment tool, as the third SHS instrument. Chinese researchers developed a self-reported questionnaire to assess teenage psychological problems [33]. The MSQA assesses uncomfortable symptoms experienced by respondents in the past three months, and includes 71 items divided into six symptom dimensions: lack of physical energy (11 items), physiological dysfunction (11 items), weakened immunity (10 items), emotional symptoms (17 items), behavioral symptoms (9 items), and social adaptation problems (13 items) [43]. Each item has six answer categories: none or last <1 week, 1 week, 2 weeks, 1 month, 2 months, and 3 months [43]. Emotional and behavioral symptoms are measured using 17 and 9 items, respectively. There are 13 items that measure social adaptation issues (e.g., “always disliked school”) [31].

#### 3.3.2. Scoring System for the MSQA

The MSQA measures emotional, behavioral, and social symptoms [31]. Summing item scores yields the final scores. Summing the 39 item scores yields psychological symptoms. The MSQA National Norm Development [33] sets the psychological symptom cut-off at the 90th percentile for all adolescents. Emotional, behavioral, social adaptability, and psychological symptoms have cut-off values of 3, 1, 4, and 8, respectively [31]. The MSQA also evaluates psychophysiological functioning. The MSQA has 39 questions on three dimensions based on symptoms experienced in the past three months: 17 for emotional symptoms (e.g., “Do you always feel nervous?”), 9 for behavioral symptoms (e.g., “Do you always have the impulse to damage something?”), and 13 for social adaptation problems (e.g., “Were you always not suited to school life?”) [44,45]. All questions include six response alternatives based on symptom duration: none, last <1 week, last 1–2 weeks, last 1 month, last 2 months, last 3 months [31]. The symptom duration “last 0–1 week” was converted into “1” (positive items) and “none or last <1 week” into “0” (negative items) [44,45,46]. Psychopathological symptoms required eight or more “1” scores [45].

#### 3.3.3. Reliability and Validity Indicators of the MSQA

We found four studies assessing the psychometric properties of the MSQA. The test–retest reliability was around 0.87 in three studies. Moreover, the Cronbach alpha coefficient and split-half reliability were around 0.96 and 0.94, respectively [33,43,44,45,47]. The total scale of Cronbach’s α for physiological, psychological, and social components demonstrated good reliability at 0.91, 0.85, and 0.85, respectively [36], as shown in Table 3.

**Table 3:** Studies on reliability and validity of the MSQA and SSS.

Instrument	Authors and Year	Sample Size	Reliability		Validity
			Internal Consistency	Test-Retest Reliability	
MSQA	Tao et al. (2008) [47]	7104 middle school student	Cronbach’s α = 0.96, split-half reliability = 0.94	0.86	SCL-90 = 0.63 and CMI the criterion-related validity = 0.64
	Yao et al. (2015) [33]	5249 students from university and high school students	Cronbach’s α = 0.96, split-half reliability coefficient = 0.94	0.87	Not Reported
	Huang et al. (2022) [44]	778 minors (<18 years old)	Cronbach’s α = 0.96	Not Reported	Not Reported
	Tang et al. (2021) [43]	15,713 students	Cronbach’s α = 0.95	Not Reported	Not Reported
SSS	[Bi, Jl., 2019] [42]	6232 students	Cronbach’s α of the total scale = 0.942, Cronbach’s α for sub-scales: Physiological = 0.915, Psychological = 0.856, and Social 0.850	Not Reported	Correlation between dimension: Physiological = 0.929, Psychological = 0.803, and Social 0.774 Bartlett test < 0.001 KMO = 0.94

MSQA = Multidimensional Sub-Health Questionnaire of Adolescents; SSS = Sub-health Self-rating Scale; Goodness of fit index (GFI); Adjusted Goodness of Fit Index (AGFI); Comparative fit index (CFI); KMO = Kaiser–Meyer–Olkin.

### 3.4. Sub-Health Self-Rating Scale (SSS)

#### 3.4.1. Description of the SSS

The search retrieved the Sub-Health Self-Rating Scale (SSS), which Chinese researchers developed to assess the SHS of university students [34]. It determines the SHS of individuals by assessing three dimensions of health (physiological, psychological, and social). A total of 58 items comprises the scale, and scoring is conducted on ten labelled factors (F1 to F10). The physiological dimension symptoms include six factors: sleep, fatigue, skin, pain, digestive, and urine, which are labelled F5, F7, F9, F3, F4, and F10, respectively. The social encompass dimension symptoms comprise two factors: F6 and F8, capability and self-respect factor, and social relationship factor, respectively. The psychological dimension symptoms contain two factors: F2—passive feeling factor, and F1—positive feeling factor [42].

#### 3.4.2. Scoring System for SSS Measures

The SSS is scored by adding the raw scores on items or sub-scales [42]. Each item contains five answer categories for symptom severity (never = 5, occasionally = 4, sometimes = 3, constantly = 2, and always = 1). Before adding the scores, the 16 symptoms are inversely converted. The converted score is the raw score minus the lowest possible sub-scale or total scale score, divided by the highest possible score minus the lowest. The T score measures test score variability as (X + −X)/S, where X is the raw score, −X is the overall mean score, and S is the population standard deviation [42].

#### 3.4.3. Reliability and Validity Indicators of the SSS

The SSS’s psychometric qualities were assessed using the Cronbach α coefficient of 0.942 [42]. The reliability for each physiological, psychological, and social dimension was reliable: 0.915, 0.856, and 0.850, respectively [42]. The Bartlett test of sphericity showed validity (2 = 7778.7; *p* = 0.000), and the Kaiser–Meyer–Olkin (KMO) score of sample adequacy was 0.94, as shown in Table 3.

To sum up, the systematic review of articles on SHS measurement tools demonstrated some evidence about the psychometric properties of instruments; most of them were based upon studies from China. Among the reliability indicators, three parameters were commonly reported: (1) the internal consistency measured by Cronbach’s α value ranged between 0.71 and 0.96; (2) the test–retest reliability ranged from 0.64 to 0.98; and (3) the split-half reliability coefficient values ranged between 0.64 and 0.98, and between 0.83 and 0.96, respectively. All three indicators of reliability revealed acceptable levels of evidence about the reliability of these measures.

The indicators of the validity of tools were determined through (1) construct validity, (2) convergent validity, and (3) divergent validity, and it was accomplished for three subjective measures, namely the SHSQ-25, MSQA, and SSS. For the validity coefficient values in the case of SHSQ-25 > 0.71, SHMS-1.0 ranged from 0.64 to 0.87, and SSS ranged from 0.74 to 0.96, and can be considered as acceptable.

## 4. Discussion

We believe this is the first systematic review of the validity and reliability of SHS instruments. We found four instruments measuring SHS, and there have been few studies conducted on the psychometric properties of these SHS instruments. Encouragingly, all instruments showed some psychometric testing, with comparatively higher coefficient values (0.70–0.98) on the reliability tests and correlation coefficient values (0.70–0.92) for the validity test.

Utilizing standardized measures to assess the SHS has many advantages. When evaluating an intervention, there is no rationale for creating new (invalidated) instruments when excellent standardized tools already can be used for free or at a minimal cost, given that these instruments were relatively good in discriminating SHS. It was imperative to scrutinize items or elements used in measuring SHS by each instrument. For example, the SHMS V1.0, which has a total of 39 items, has 4 separate items used to make general health self-evaluations, with the remaining 35 divided among three symptom dimensions. The first dimension comprises 14 items that assess physiological symptoms; the second dimension includes 12 items and assesses psychological symptoms; and the third dimension consists of 9 items that assess social symptoms [31,36,37,40,41,46]. The SHMS V 1.0’s structural validity indicates a strong association between question scores and dimensional scores of 0.656 to 0.878. Items, dimensions, and sub-scales are also associated, since dimension scores range from 0.586 to 0.868 [42].

On the other hand, the SSS instrument had a good correlation coefficient on the total scale and the sub-scales. Each question had a correlation of 0.52–0.89 for physiological, psychological, and social dimensions [36]. The questionnaire is brief and valid. Thus, it is acceptable to use for undergraduate students [36]. The self-rating scale may also misdiagnose psychological and social mental diseases as SHS [36].

Interestingly, the SHSQ-25, which has 25 items in five domains, has been validated in Chinese, African, and European populations [5,16,29]. The SHSQ-25 screens multidimensional health constructs to identify poor health status and chronic stress. The SHSQ-25 sub-scales overlapped in the Ghanaian population, leading to redesigning the three-factor structure into fatigue, immune-cardiovascular, and cognitive sub-scales [29]. The SHSQ-25 was found to be a reliable tool because of good internal consistency of the Cronbach’s α values (0.70–0.95) for all sub-scales [4]. On the other hand, Wang and Yan [15] found higher coefficient values (0.91) for test–retest reliability among 3000 adults. Moreover, in an effort to assess construct validity, Adua et al. [29] found a construct validity threshold >0.7. These findings demonstrate that the SHSQ-25 is a valuable instrument for assessing SHS, given the reliability and validity, shortness, and ease to manage [5,14,23]; therefore, if translated into different local languages, the SHSQ-25 can easily be implemented by lay persons. Several studies have confirmed the ability of the SHS Q-25 to capture high-risk groups with poor lifestyle behaviors, chronic disease (T2DM, CVD), and biochemical and molecular abnormalities [48]. Furthermore, in resource-constrained countries, the instrument could be recommended as a screening tool for the early detection of chronic diseases.

Additionally, the MSQA, designed to assess uncomfortable symptoms experienced by respondents, had 71 questions divided into six symptom dimensions [43]. The MSQA, which has been purely used in high school and university students, has been confirmed to have higher internal consistency Cronbach’s α coefficients (0.87–0.96) [36,43,44,45,46,47]. Zhang et al. [46] found alpha coefficient values of 0.74–0.88 for the six sub-scales, indicating good internal consistency. However, we did not find a study that assessed this instrument’s validity in English. Given the good reliability scores for the MSQA, it is suitable for assessing the psychological symptoms of adolescents. However, the number of questions and their target population may limit its application in adolescents.

In short, comments on the validity of individual health and well-being tools were based on articles that evaluated or reported on the validity of the measurement tools. These articles’ findings were taken at face value. However, many of the articles did not use standard terms for validity, and did not evaluate validity in the same way. Even though some tools have limitations, the full table was provided for information. The scoring has some subjective parts, and different criteria for scoring could be used to come up with different total scores and rankings. However, we used a logical framework to separate the tools that could be used to evaluate SHS in community interventions. With the information in this paper, researchers and clinicians can find and use instruments that have real proof in possessing appropriate psychometric properties to measure concepts that are relevant to their goals.

## 5. Conclusions

In conclusion, four instruments for SHS were found through our systematic analyses, and their psychometric properties were found to be adequate. Since they have been proven to be reliable and valid, are easy to use, and often have established population norms, and they are good choices. However, the SHSQ-25 was found to be better for the general population than the SHMS V1.0, MSQA, and SSS as a result of the length and population-specific focus of the SHMS V1.0, MSQA, and SSS. In contrast, the SHSQ-25 is brief and simple to complete, and its psychometric qualities have been examined in numerous populations around the world. Therefore, it is important to adapt the SHSQ-25 and translate it into various languages, such as Arabic, in order to evaluate out how they perform in the general populations of other countries.

## Figures and Tables

**Table 1:** Current gaps in previous systematic reviews on SHS.

Authors and Year	Title	Aims	Gap
Zhang Y., et al. (2015) [3]	A systemic review of suboptimal health	Analyze the currently available SHS research and discuss the related issues about its concept and diagnostic criteria. Differentiate between chronic fatigue syndrome and psychosomatic diseases.	Analyzed and discussed the related issues about the concept of SHS and the diagnostic criteria; however, did not focus in the psychometric properties of SHS instruments.
Zhong et al. (2010) [5]	A literature review on the conceptual framework of sub-health	Assess conceptual framework, diagnostic criteria, and their operability; foundational support of SHS conditions.	Focused on articulating the conceptual framework of SHS, but never addressed issues related to SHS instruments.
Wei Wang (2021) [24]	A joint position paper of the SHS Study Consortium and European Association for PPPM	Demonstrate advanced strategies in bio/medical sciences and healthcare through focused on SHS conditions in PPPM; for potential benefits in healthcare systems, to improved life quality, and advanced professionalism of healthcare givers, and a sustainable healthcare economy.	Focused on the assessment of SHS for PPPM; however, did not adopt a systematic review approach to assess the psychometric properties of tools.

## Data Availability

Not applicable.

## Supplementary Materials

The following supporting information can be downloaded at: https://www.mdpi.com/article/10.3390/jpm13020299/s1, The search filters and indicators set can be retrieved from Supplementary Files SI and SII.
