# Supplementary material for: Psychometric Properties of Suboptimal Health Status Instruments: A Systematic Review
Source: J Pers Med. 2023 Feb 8;13(2):299. doi: 10.3390/jpm13020299 (PMC9967625; doi:10.3390/jpm13020299)
Supplement: Supplementary file 1 [file jpm-13-00299-s001.zip › File S1.pdf]

Search Strategy and Filters

#1 **Construct search**: The construct search should be defined by the user. It includes search terms for the construct to be measured. For example, quality of life, physical activity, etc.

Filter	Results
(("suboptimal"[All Fields] OR "suboptimally"[All Fields]) AND ("health status"[MeSH Terms] OR ("health"[All Fields] AND "status"[All Fields]) OR "health status	4112
"Suboptimal"[Title/Abstract] AND "Health"[Title/Abstract] AND "Status"[Title/Abstract])	957
(((suboptimal) AND (health)) AND (status)) AND (instrument) Filters: Abstract, Free full text, Full text	55 results

#2 **population search**: The population search should also be defined by the user. It includes search terms for the population of interest. For example: children, diabetes, etc.

("Women"[Title/Abstract]) AND ("Suboptimal Health Status"[Title/Abstract])	10 results
(Men) AND ("Suboptimal health status")	8 results

#3 **instrument search:** The instrument search should also be defined by the user. It includes search terms for the instruments of interest. For example: questionnaires, performance-based tests, etc.

("Suboptimal health status") AND ("Survey")	24 results
("suboptimal health status") AND ("scale")	22 results

#4 **#1 AND #2 AND #3 AND measurement properties filter**: These searches should then be combined with the search filter for measurement properties to find all studies on the measurement properties of the instruments of interest that measure the construct of interest in the population of interest. This should be done by replacing the term measurement properties filter above by all terms of the measurement properties filter (copy the entire filter exactly at this place).

I used (("suboptimal health") AND ("scale")) along with suggested filter for this	25 results

#5 #4 NOT exclusion filter: The exclusion filter is meant to remove irrelevant records from the search, such as case reports and animal studies. Again, this should be done by replacing the term exclusion filter above by all terms of the exclusion filter (copy the entire filter exactly at this place).

I used Exclusion Filter given below along with above filters Measurement properties filter and suboptimal health status
Results Found with this filter = 35

Measurement properties filter:

(instrumentation[sh] OR methods[sh] OR "Validation Studies"[pt] OR "Comparative Study"[pt] OR "psychometrics"[MeSH] OR psychometr*[tiab] OR clinimetr*[tw] OR clinometr*[tw] OR "outcome assessment (health care)"[MeSH] OR "outcome assessment"[tiab] OR "outcome measure*"[tw] OR "observer variation"[MeSH] OR "observer variation"[tiab] OR "Health Status Indicators"[Mesh] OR "reproducibility of results"[MeSH] OR reproducib*[tiab] OR "discriminant analysis"[MeSH] OR reliab*[tiab] OR unreliab*[tiab] OR valid*[tiab] OR "coefficient of variation"[tiab] OR coefficient[tiab] OR homogeneity[tiab] OR homogeneous[tiab] OR "internal consistency"[tiab] OR (cronbach*[tiab] AND (alpha[tiab] OR alphas[tiab])) OR (item[tiab] AND (correlation*[tiab] OR selection*[tiab] OR reduction*[tiab])) OR agreement[tw] OR precision[tw] OR imprecision[tw] OR "precise values"[tw] OR test-retest[tiab] OR (test[tiab] AND retest[tiab]) OR (reliab*[tiab] AND (test[tiab] OR retest[tiab])) OR stability[tiab] OR interrater[tiab] OR inter-rater[tiab] OR intrarater[tiab] OR intra-rater[tiab] OR intertester[tiab] OR inter-tester[tiab] OR intratester[tiab] OR intra-tester[tiab] OR interobserver[tiab] OR inter-observer[tiab] OR intraobserver[tiab] OR intra-observer[tiab] OR intertechnician[tiab] OR inter-technician[tiab] OR intratechnician[tiab] OR intra-technician[tiab] OR interexaminer[tiab] OR inter-examiner[tiab] OR intraexaminer[tiab] OR intra-examiner[tiab] OR interassay[tiab] OR inter-assay[tiab] OR intraassay[tiab] OR intra-assay[tiab] OR interindividual[tiab] OR inter-individual[tiab] OR intraindividual[tiab] OR intra-individual[tiab] OR interparticipant[tiab] OR inter-participant[tiab] OR intraparticipant[tiab] OR intra-participant[tiab] OR kappa[tiab] OR kappa's[tiab] OR kappas[tiab] OR repeatab*[tw] OR ((replicab*[tw] OR repeated[tw]) AND (measure[tw] OR measures[tw] OR findings[tw] OR result[tw] OR results[tw] OR test[tw] OR tests[tw])) OR generaliza*[tiab] OR generalisa*[tiab] OR concordance[tiab] OR (intraclass[tiab] AND correlation*[tiab]) OR discriminative[tiab] OR "known group"[tiab] OR "factor analysis"[tiab] OR "factor analyses"[tiab] OR "factor structure"[tiab] OR "factor structures"[tiab] OR dimension*[tiab] OR subscale*[tiab] OR (multitrait[tiab] AND scaling[tiab] AND (analysis[tiab] OR analyses[tiab])) OR "item discriminant"[tiab] OR "interscale correlation*"[tiab] OR error[tiab] OR errors[tiab] OR "individual variability"[tiab] OR "interval variability"[tiab] OR "rate variability"[tiab] OR (variability[tiab] AND (analysis[tiab] OR values[tiab])) OR (uncertainty[tiab] AND

(measurement[tiab] OR measuring[tiab])) OR “standard error of measurement”[tiab] OR sensitiv*[tiab] OR responsive*[tiab] OR (limit[tiab] AND detection[tiab]) OR “minimal detectable concentration”[tiab] OR interpretab*[tiab] OR ((minimal[tiab] OR minimally[tiab] OR clinical[tiab] OR clinically[tiab]) AND (important[tiab] OR significant[tiab] OR detectable[tiab]) AND (change[tiab] OR difference[tiab])) OR (small*[tiab] AND (real[tiab] OR detectable[tiab]) AND (change[tiab] OR difference[tiab])) OR “meaningful change”[tiab] OR “ceiling effect”[tiab] OR “floor effect”[tiab] OR “Item response model”[tiab] OR IRT[tiab] OR Rasch[tiab] OR “Differential item functioning”[tiab] OR DIF[tiab] OR “computer adaptive testing”[tiab] OR “item bank”[tiab] OR “cross-cultural equivalence”[tiab])

Exclusion filter

(“addresses”[Publication Type] OR “biography”[Publication Type] OR “case reports”[Publication Type] OR “comment”[Publication Type] OR “directory”[Publication Type] OR “editorial”[Publication Type] OR “festschrift”[Publication Type] OR “interview”[Publication Type] OR “lectures”[Publication Type] OR “legal cases”[Publication Type] OR “legislation”[Publication Type] OR “letter”[Publication Type] OR “news”[Publication Type] OR “newspaper article”[Publication Type] OR “patient education handout”[Publication Type] OR “popular works”[Publication Type] OR “congresses”[Publication Type] OR “consensus development conference”[Publication Type] OR “consensus development conference, nih”[Publication Type] OR “practice guideline”[Publication Type]) NOT (“animals”[MeSH Terms] NOT “humans”[MeSH Terms])
