# Supplementary material for: Psychometric Properties of Suboptimal Health Status Instruments: A Systematic Review
Source: J Pers Med. 2023 Feb 8;13(2):299. doi: 10.3390/jpm13020299 (PMC9967625; doi:10.3390/jpm13020299)
Supplement: Supplementary file 1 [file jpm-13-00299-s001.zip › File S2.pdf]

**COSMIN definitions of domains, measurement properties, and aspects of measurement properties**

| Term | | | Definition |
|---|---|---|---|
| **Domain** | | An aspect of a measurement property | |
| **Reliability** | | | The degree to which the measurement is free from measurement error |
| **Reliability (extended definition)** | | | The extent to which scores for patients who have not changed are the same for repeated measurement under several conditions: e.g. using different sets of items from the same health-related-patient reported outcomes (HR-PRO) (internal consistency); over time (test-retest); by different persons on the same occasion (inter-rater); or by the same persons (i.e. raters or responders on different occasions intra-rater |
| | Internal consistency | | The degree of interrelatedness among the items |
| | Reliability | | The proportion of the total variance in the measurements which is due to 'true* differences between patients |
| | Measurement error | | The systematic and random error of a patient's score that is not attributed to true changes in the construct to be measured |
| **Validity** | | | The degree to which an HR-PRO instrument measures the construct s it ur orts to measure |
| | Content validity | | The degree to which the content of an HR-PRO instrument is an adequate reflection of the construct to be measured |
| | | Face validity | The degree to which (the items of) an HR-PRO instrument indeed looks as though they are an adequate reflection of the construct to be measured |
| | Construct validity | | The degree to which the scores of an HR-PRO instrument are consistent with hypotheses (for instance with regard to internal relationships, relationships to scores of other instruments, or differences between relevant groups) based on the assumption that the HRPRO instrument validly measures the construct to be measured |

| | | | |
|---|---|---|---|
| | | Structural validity | The degree to which the scores of an HR-PRO instrument are an adequate reflection of the dimensionality of the construct to be measured |
| | | Hypotheses testing | Idem construct validity |
| | | Cross-cultural validity | The degree to which the performance of the items on a translated or culturally adapted HR-PRO instrument is an adequate reflection of the performance of the items of the original version of the HR-PRO instrument |
| | Criterion validity | | The degree to which the scores of an HR-PRO instrument are an adequate reflection of a 'gold standard'. |
| **Responsiveness** | | | The ability of an HR-PRO instrument to detect change over time in the construct to be measured |
| | Responsiveness | | Idem responsiveness |
| **Interpretability*** | | | Interpretability is the degree to which one can assign qualitative meaning - that is, clinical or commonly understood connotations — to an instrument's quantitative scores or change in scores. |

The word 'truth must be seen in the context of the CTT, which states that any observation is composed of two components — a true score and an error associated with the observation. 'True' is the average score that would be obtained if the scale were given an infinite number of times. It refers only to the consistency of the score, and not to its accuracy (ref Streiner & Norman)

* Interpretability is not considered a measurement property, but an important characteristic of a measurement instrument
